# Interferon regulatory factor-1 regulates cisplatin-induced apoptosis and autophagy in A549 lung cancer cells

**DOI:** 10.1007/s12032-021-01638-z

**Published:** 2022-01-29

**Authors:** Lemeng Zhang, Tianli Cheng, Hua Yang, Jianhua Chen, Xiaoping Wen, Zhou Jiang, Huihuang Yi, Yongzhong Luo

**Affiliations:** Thoracic Medicine Department 1, Hunan Cancer Hospital, Tongzipo Rd 283#, Yuelu District, Changsha, 410013 Hunan Province People’s Republic of China

**Keywords:** NSCLC, Cisplatin, Apoptosis, Autophagy, IRF-1

## Abstract

This study aimed to investigate the expression and function of interferon regulatory factor-1 (IRF-1) in non-small cell lung cancer (NSCLC). IRF-1 expression and its prognostic value were investigated through bioinformatic analysis. The protein expression levels of IRF-1, cleaved caspase 3, and LC3-I/II were analyzed by western blotting. A lentiviral vector was used to overexpress or knockdown IRF-1 in vitro. Mitochondrial membrane potential (MMP) and reactive oxygen species (ROS) were analyzed by JC-1 and DCFH-DA staining, respectively. ATP, SOD, MDA, cell viability, LDH release, and caspase 3 activity were evaluated using commercial kits. Compared to the levels in normal tissues, IRF-1 expression was significantly lower in lung cancer tissues and was a prognostic factor for NSCLC. Cisplatin treatment-induced IRF-1 activation, ROS production, ATP depletion, SOD consumption, and MDA accumulation in A549 lung cancer cells. IRF-1 overexpression promoted mitochondrial depolarization, oxidative stress, and apoptotic cell death and inhibited autophagy in A549 cells, and these effects could be reversed by IRF-1 knockdown. These data suggest that IRF-1 regulates apoptosis, autophagy and oxidative stress, which might be served as a potential target for increasing chemotherapy sensitivity of lung cancer.

## Background

Lung cancer is a malignant disease with the highest morbidity and mortality worldwide, as it accounts for 18% of cancer-associated deaths [1]. Approximately 80% of lung cancers are non-small cell lung cancer (NSCLC) [2]. Although significant progress has been made in targeted therapy and immunotherapy for NSCLC [3, 4], the prognosis remains poor, and the 5-year survival rate of patients with advanced disease is less than 15% [5].

Cisplatin is the first-line chemotherapy drug for NSCLC [6]. Re-sensitizing resistant tumors to cisplatin remains a major challenge in clinical treatment. The main mechanism of cisplatin-induced cell death is apoptosis [7, 8], and inhibitors of apoptosis proteins drive cisplatin resistance [9]. Autophagy is also involved in cisplatin resistance [10], and it has been suggested that activation of autophagy promotes cisplatin resistance in ovarian cancer [11]. In renal cell carcinoma cells, mitochondrial depolarization and reactive oxygen species (ROS) production were shown to be involved in resistance to chemotherapy [12], sorafenib-induced depolarization, and ROS accumulation, which in turn activates caspase-8, an important regulator of apoptosis. Han et al. reported that mitochondrial fission-induced cisplatin resistance in hypoxic ovarian cancer cells through ROS, while inhibition of mitochondrial fission increased cisplatin sensitivity [13].

Interferon regulatory factor-1 (IRF-1) is a nuclear transcription factor that plays key roles in interferon expression, lymphocyte growth and differentiation, innate and acquired immunity, and other immune-related events [14, 15]. Recent studies have implicated IRF-1 in the pathogenesis of malignant diseases. Deletions or mutations in *IRF-1* have been found in numerous human tumor tissues, including myelodysplastic syndrome [14], leukemia [16], gastric cancer [17] and breast cancer. In addition, IRF-1 mediates apoptosis in ovarian cancer [18] and breast cancer [19] and regulates autophagy in breast cancer cells [20], splenocytes [21] and macrophages [22], which are cell fate determinants. Interestingly, IRF-1 could reverse the multiple drug resistance of gastric cancer by decreasing the expression of P-glycoprotein [23], and high expression of IRF-1 contributes to 5-fluorouracil chemosensitivity in gastric cancer cells [24]. Nevertheless, the activation status and function of IRF-1 in lung cancer and cisplatin resistance have not been reported.

In the current study, we first explored the expression of IRF-1 in lung cancer tissues using public databases and clinical samples. Next, we observed cisplatin-induced IRF-1 activation and explored its effects on mitochondrial homeostasis, autophagy, and apoptosis in lung cancer cells. A comprehensive, in-depth understanding of the activity and mechanism of IRF-1 in NSCLC is of theoretical and practical significance for the treatment of lung cancer.

## Methods

### Analysis of IRF-1 expression in silico

GEPIA [25] (http://gepia.cancer-pku.cn/index.html) was utilized to explore the expression of IRF-1 in different tumors in the cancer genome atlas (TCGA) database. Scatter plots and histograms were drawn, and survival analyses were conducted. Expression data and corresponding clinical information for lung adenocarcinoma (LUAD) and lung squamous cell carcinoma (LUSC) in TCGA database were acquired using UCSC Xena (http://xena.ucsc.edu/) [26]. The original count values were converted to transcripts per million (TPM) using TPM standardization. The extracted IRF-1 values were used for further analysis. IRF-1 protein expression levels in LUAD and LUSC were verified using the Human Protein Atlas (HPA) [27], which is designed for the investigation of protein expression in various human tissues and organs.

### Reagents and antibodies

Cisplatin, methyl thiazolyl tetrazolium (MTT), and LDH-dependent cytotoxic nonradioactive cytotoxicity assays were obtained from Promega (Madison, WI, USA). The LC3B antibody was purchased from Novus Biologicals (Littleton, CO, USA). *N*-acetylcysteine (NAC), β-actin, and 2,7-dichloro-fluorescin diacetate (DCFH-DA) were purchased from Sigma-Aldrich (St. Louis, MO, USA). An anti-IRF-1 antibody was obtained from Santa Cruz Biotechnology (Santa Cruz, CA, USA). The Caspase 3 Fluorometric Assay kit was obtained from BioVision (Milpitas, CA, USA). Goat anti-rabbit secondary antibodies were purchased from Thermo Fisher Scientific Inc. (Waltham, MA, USA). 5,5′,6,6′-Tetrachloro-1,1′,3,3′-tetraethyl-benzimidazolylcarbocyanine iodide (JC-1) was obtained from Invitrogen (Carlsbad, CA, USA). Assay kits for ATP, superoxide dismutase (SOD), and malondialdehyde (MDA) were obtained from Nanjing Jiancheng Bioengineering Institute. (Nanjing, China). The FITC-Annexin V/7-AAD apoptosis detection kit was obtained from BD Biosciences (USA).

### Clinical sample collection

This study included lung cancer patients who underwent surgery at Hunan Cancer Hospital (Changsha, Hunan, China) between August 2014 and August 2015. All diagnoses were confirmed from histopathological reports. The Ethics Committee of Hunan Cancer Hospital approved this study, and informed consent was obtained from all study patients.

### Cell culture

The human lung cancer cell lines A549, SK-MES-1, H1299, H460, H358, and H1975 were obtained from American Type Culture Collection (Manassas, VA, USA). All cells were cultured in PRMI-1640 or DMEM supplemented with 10% FBS (Biowest, South America Origin), 100 U/ml penicillin sodium, and 100 mg/mL streptomycin sulfate at 37 °C in a humidified atmosphere containing 5% CO_2_, as previously described [28].

### Cell transfection

IRF-1 shRNA and overexpression lentiviral vectors (Hanyin, Shanghai, China) were prepared at a titer of 1 × 10^9^ TU/mL. These vectors were transfected into cells using 5 µg/mL polyamine in RMI-1640 medium at a multiplicity of infection (MOI) of 20:1. After 4 h of transfection, the cells were cultured for 48 h in fresh culture medium. The efficiency of IRF-1 knockdown and overexpression was assessed by western blotting.

### Western blotting

Following treatment with cisplatin (20 µM), cytoplasmic and nuclear proteins were extracted according to the manufacturer’s instructions. Then, the proteins were separated by electrophoresis and transferred to Trans-Blot nitrocellulose membranes (Bio-Rad Laboratories). After blocking with 5% nonfat milk for 1 h at room temperature and incubation with the primary antibodies and the HRP-conjugated secondary antibody, the color reaction was initiated using the Super Signal West Pico chemiluminescent kit (Pierce Chemical Co.), and the blot was exposed to film (Eastman Kodak). The data were analyzed with densitometry using Image J software (Bethesda, MD, USA). Histone was used as an internal loading control.

### Mitochondrial membrane potential and reactive oxygen species detection

As described in a previous study [14], mitochondrial membrane potential was measured using JC-1 staining, and ROS was detected using DCFH-DA staining.

### Detection of intracellular ATP, SOD, and MDA

Intracellular ATP, MDA, and SOD levels were detected using corresponding assay kits according to the manufacturer’s instructions.

### Cell viability detection and lactate dehydrogenase release

The MTT assay was conducted to measure cell viability, and lactate dehydrogenase (LDH) was determined using the LDH-dependent Cytotoxic Non-Radioactive Cytotoxicity Assay kit, as described by Chen et al. [29].

### Caspase 3 activity assay and apoptotic cell death detection by flow cytometry

Caspase 3 activity was measured using the Caspase 3 Fluorometric Assay kit according to the manufacturer’s instructions. Apoptotic cells were detected using the FITC-Annexin V/7-AAD apoptosis detection kit according to the manufacturer’s instructions.

### Statistical analysis

Data were processed using SPSS software (version 23.0). Quantitative data are shown as the mean ± standard deviation of three replicates as determined with Student’s t-test or one-way analysis of variance (ANOVA) between two or more groups. Repeated measures ANOVA followed by an LSD test was used to analyze differences within groups. Samples from TCGA were categorized into high- and low-expression groups based on the upper and lower quartiles of IFR-1 expression. Kaplan–Meier curves and log-rank tests were used to analyze the correlation between IRF-1 expression and patient survival. Clinical phenotypes between groups were compared using the Chi-square test. Univariate Cox regression analysis was used to screen survival-related factors. A P value less than 0.05 was considered statistically significant.

## Results

### IRF-1 is depleted in non-small cell lung cancer

**Fig. 1:**
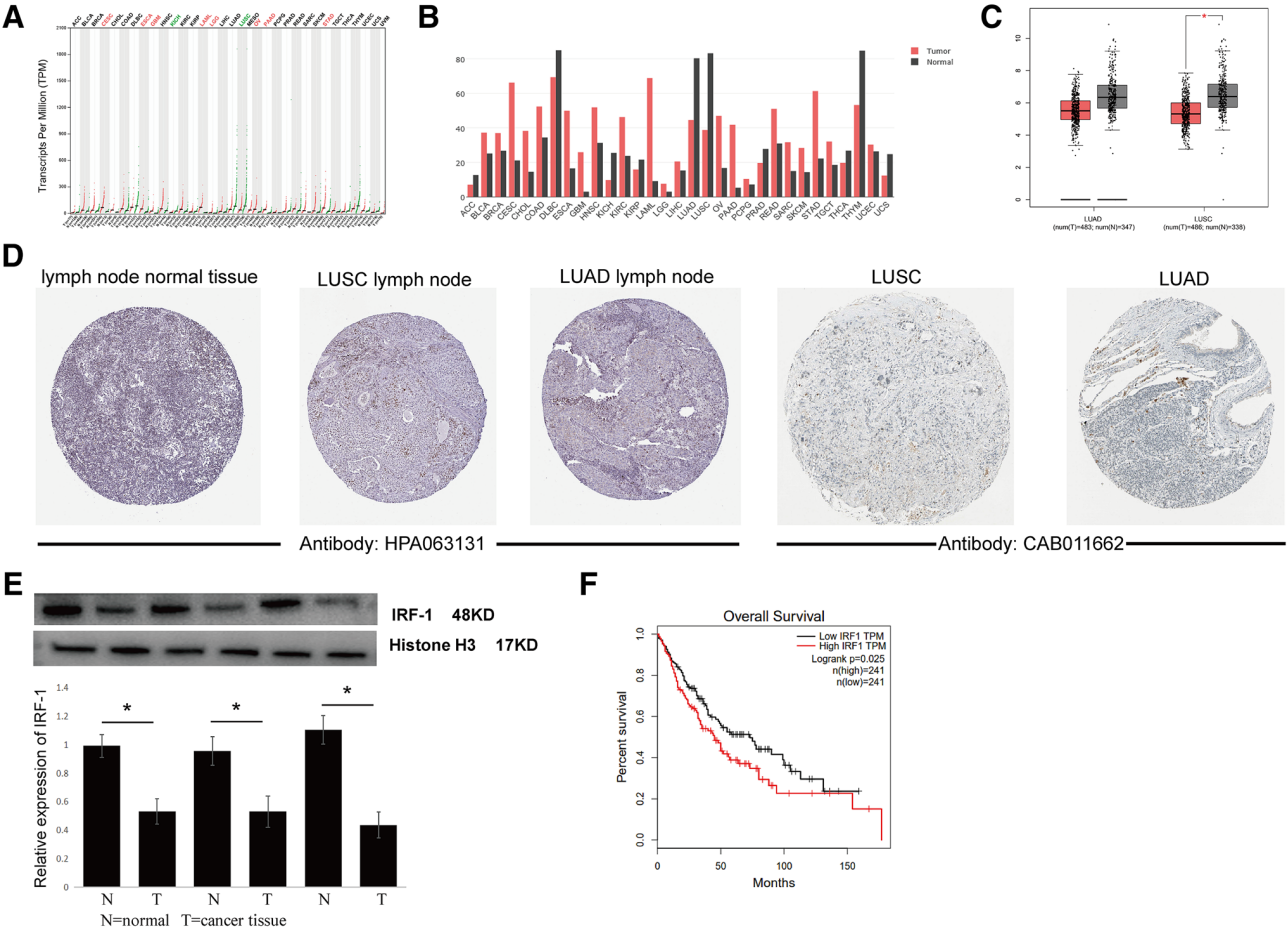
IRF-1 expression in lung cancer: TCGA database, clinical samples, and A549 cells. **A** Scatter plot showing pan-tumor IRF-1 expression profiles with paired normal tissues as analyzed by GEPIA. Each point represents the IRF-1 expression in one sample; **B** Bar plot showing the pan-tumor IRF-1 expression profiles with paired normal tissues as analyzed by GEPIA. The height of the columns represents the median IRF-2 expression in tumor and normal tissues; **C** Boxplot showing decreased IRF-1 expression levels in LUAD and LUSC samples compared to the normal controls; **D** IRF-1 immunohistochemical staining data from the HPA database; E, IRF-1 protein expression in NSCLC clinical samples as determined by western blotting; **F** Kaplan–Meier survival curve showing the prognostic value of IRF-1 expression in NSCLC in a TCGA dataset; IRF-1 protein expression in A549 cells after cisplatin (20 µM) treatment for 8 h as determined by western blotting

We used GEPIA for a pan-tumor analysis of IRF-1 expression, which showed that IRF-1 was differentially expressed in cancer tissues and its expression was inconsistent in different cancer tissues. IRF-1 expression was downregulated in adrenocortical carcinoma (ACC), kidney chromophobe (KICH), LUAD, and LUSC (Fig. 1A , B). Of particular note, IRF-1 expression was downregulated in LUAD and LUSC samples compared to the expression in normal samples (Fig. 1C). Analysis of the HPA database further verified the low IRF-1 protein levels in LUAD and LUSC, and the results indicated that IRF-1 protein levels were decreased in lung cancer tissues and lymph nodes compared to the levels in normal lymph nodes (Fig. 1D). In addition, western blot analysis of IRF-1 in clinical lung cancer tissues showed that IRF-1 expression was significantly downregulated in cancer tissues compared to the levels in normal tissues (Fig. 1E). Therefore, we concluded that IRF-1 was depleted in NSCLC tissues.

### IRF-1 is a prognostic factor

We further investigated the clinical value of IRF-1 in NSCLC using clinical data from TCGA. Table 1 shows the clinical information of patients obtained from TCGA. Based on the upper and lower quartiles of IFR-1 expression, the samples were grouped into high- and low-expression groups. There was no significant difference in the clinical characteristics between the high and low IRF-1 groups. Survival analysis indicated that IRF-1 expression was significantly correlated with prognosis (*P* < 0.05) (Fig. 1F). Univariate Cox regression analysis showed that the response to chemotherapy (HR = 0.233, *P* = 0.009), metastasis (HR = 2.129, *P* < 0.001), lymph node status (HR = 2.637, *P* < 0.001), and tumor stage (HR = 2.278, *P* < 0.001) had significant impacts on patient prognosis. In addition to these clinical factors, IRF-1 was also associated with patient prognosis, with an HR of 1.003 (95% CI 1.000–1.005, *P* = 0.031) (Table 2).

**Table 1:** Correlation between IRF-1 expression and clinical parameters

Clinical features	*N*	IRF-1		*P* value
		Low expression	High expression	
Age (years)				
< = 65	188	136	52	0.344
> 65	299	229	70	
Gender				
Female	129	92	37	0.315
Male	367	280	87	
Metastasis				
M0	407	303	104	0.2
M1	7	7	0	
Lymph node status				
N0	316	240	76	0.563
N1 + 2 + 3	175	128	47	
Tumor stage				
T1 + T2	403	303	100	0.947
T3 + T4	93	69	24

**Table 2:** Cox regression analysis of prognostic factors

	HR	95% CI	*P* value
Age (years)	1.221	0.907–1.642	0.188
Gender	1.048	0.782–1.406	0.752
IRF-1 expression	1.003	1.000–1.005	0.031*
Chemotherapy response	0.233	0.078–0.698	0.009*
Metastasis	2.129	1.243–3.648	< 0.001*
Lymph node status	2.637	1.957–3.553	< 0.001*
Tumor stage	2.278	1.547–3.353	< 0.001*

*P value < 0.05 was considered as a significant difference

### *Cisplatin induces IRF-1 activation *in vitro

**Fig. 2:**
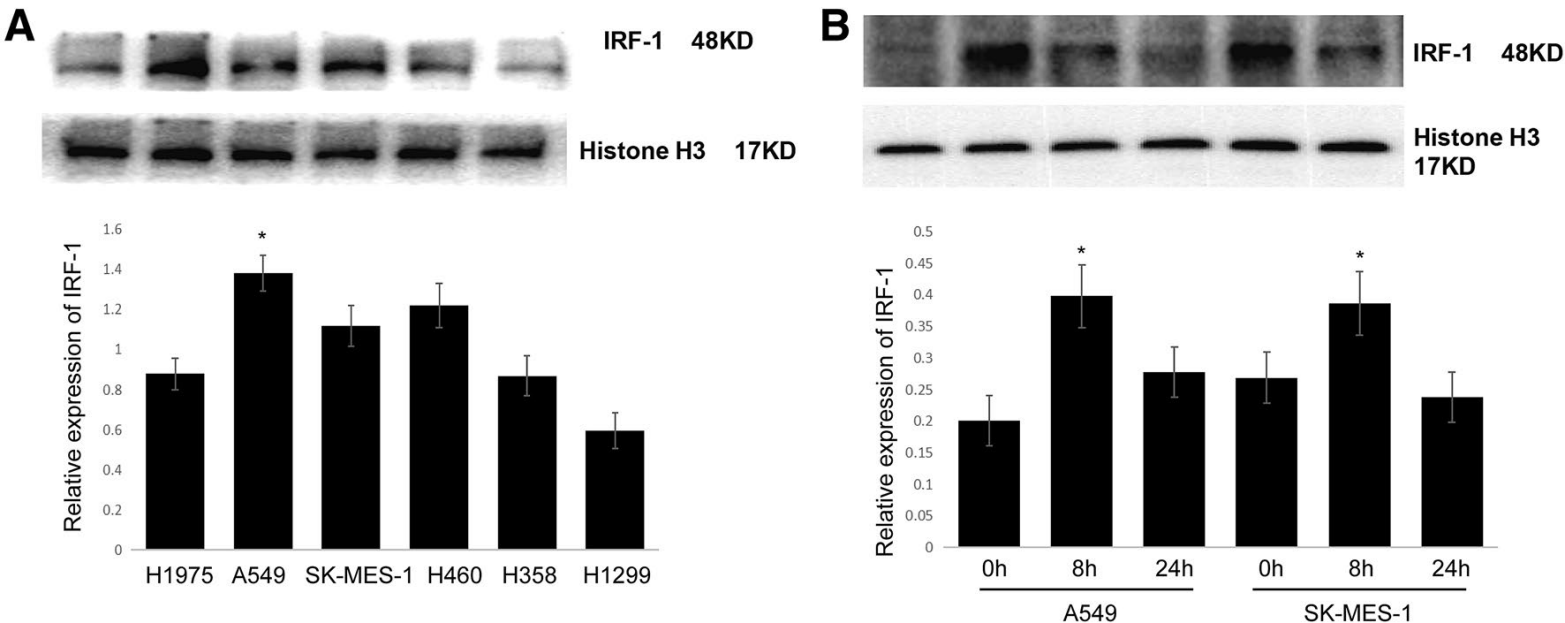
Cisplatin induces IRF-1 activation in vitro. **A** IRF-1 protein expression in different lung cancer cell lines after cisplatin (20 µM) treatment as determined by Western blotting; **B** IRF-1 protein expression in A549 and SK-MES-1 cells at 0, 8, and 24 h after cisplatin (20 µM) treatment

Cisplatin-induced IRF-1 activation was assessed in different lung cancer cell lines by Western blotting. As shown in Fig. 2A, cisplatin-induced IRF-1 activation differently in the tested cell lines; cisplatin-induced IRF-1 activation only in A549, SK-MES-1, and H460 cells, but not in the other tested lung cancer cells. Additionally, cisplatin-induced IRF-1 activation in A549 cells reached a peak at 8 h and returned to baseline at 24 h after cisplatin stimulation (Fig. 2B), demonstrating that IRF-1 levels were elevated by cisplatin in a time-dependent manner. Since the A549 cell line showed the highest levels of IRF-1 transcription and expression following cisplatin treatment, it was chosen for further study.

### Cisplatin induces ROS production and mitochondrial depolarization

**Fig. 3:**
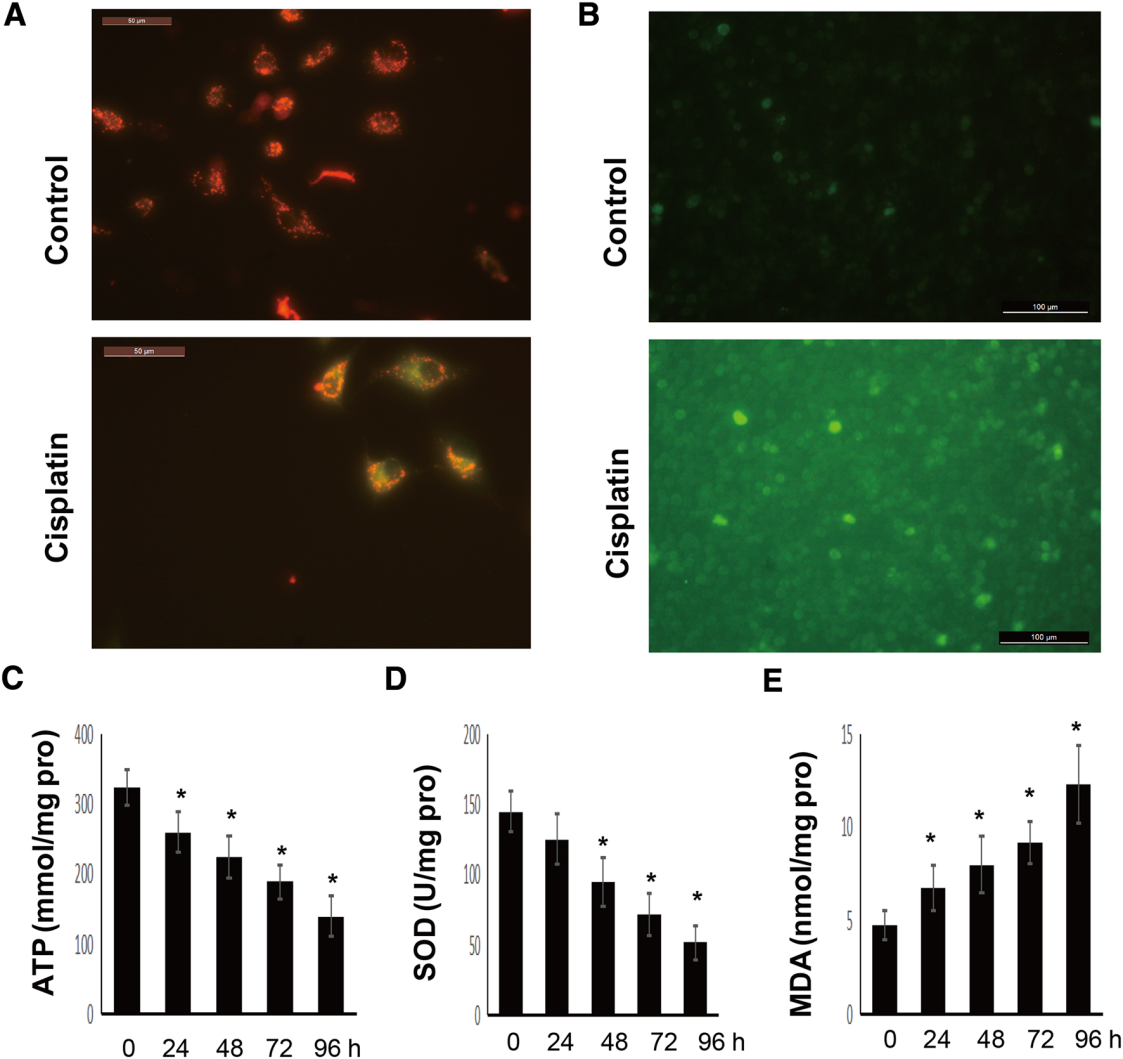
Cisplatin treatment leads to mitochondrial depolarization and oxidative stress. **A** Mitochondrial membrane potential as determined by JC-1 staining; **B** ROS detection by DCFH-DA staining in A549 cells treated with 20 µM cisplatin for 8 h, and the levels of ATP (**C**), SOD (**D**), and MDA (**E**) in A549 cells treated with 20 µM cisplatin at different time points. *P < 0.05 vs. the control

Compared with control cells, cisplatin-treated A549 cells showed lower mitochondrial membrane potential (Fig. 3A) and increased ROS production (Fig. 3B), suggesting that cisplatin treatment leads to mitochondrial depolarization and oxidative stress in A549 cells. We next determined the effects of cisplatin on intracellular ATP levels and oxidative stress-related biochemical indices, i.e., SOD and MDA levels. As shown in Fig. 3C–E, cisplatin treatment significantly promoted ATP depletion in A549 cells in a time-dependent manner. Similarly, cisplatin treatment significantly increased SOD consumption and MDA accumulation in A549 cells in a time-dependent manner.

### IRF-1 overexpression promotes mitochondrial depolarization and oxidative stress

**Fig. 4:**
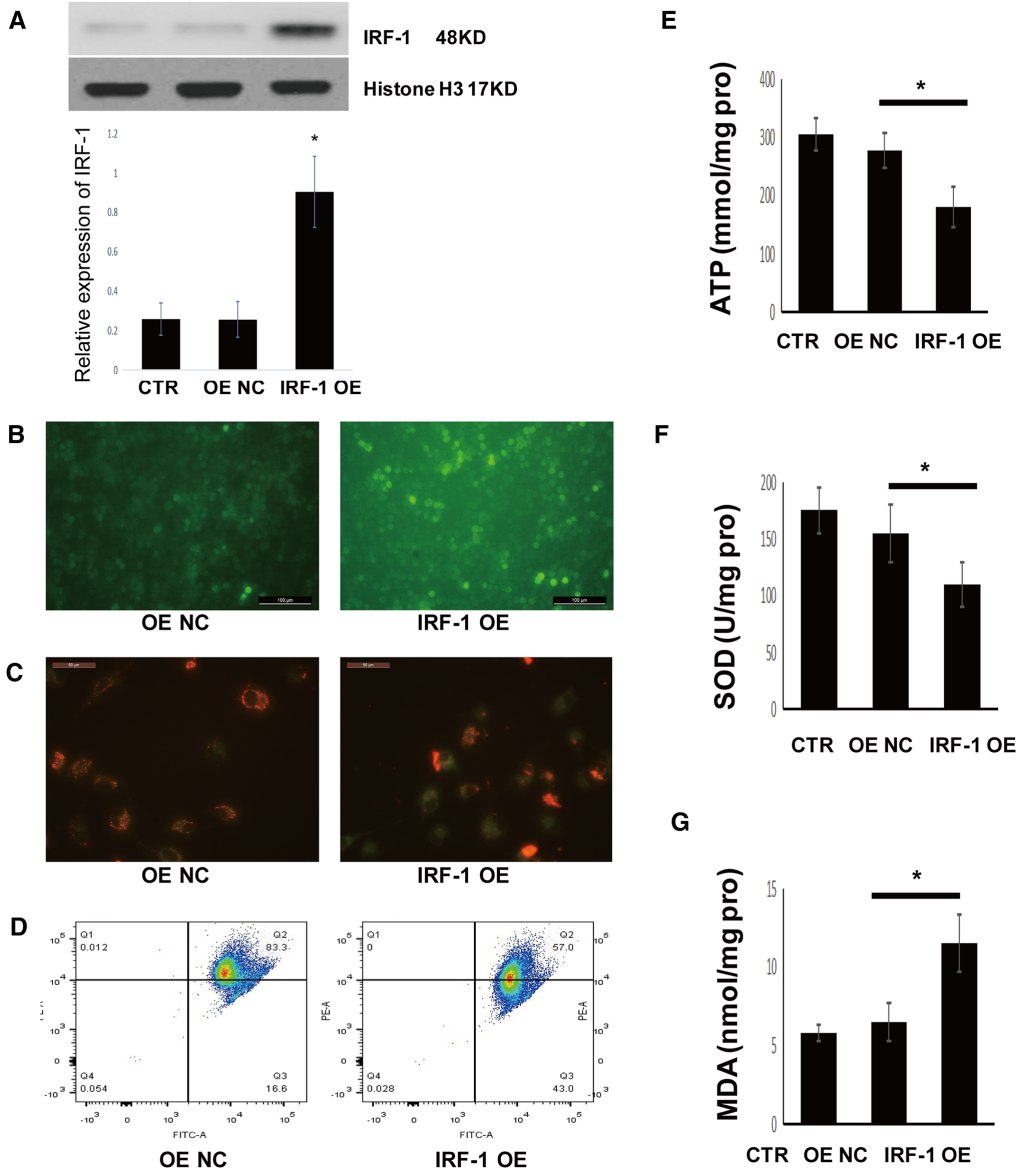
IRF-1 overexpression promotes mitochondrial depolarization and oxidative stress. **A** IRF-1 protein expression as determined by western blotting; **B** ROS release as determined by DCFH-DA staining; **C** mitochondrial membrane potential as determined by JC-1 staining; **D** apoptotic cell death as detected by flow cytometry, and ATP (**E**), SOD (**F**), and MDA (**G**) levels as determined with respective test kits. *P < 0.05 vs. the control. CTR, control; NC, negative control; OE, overexpression; OE NC, negative control of overexpression group, using empty vector

To study the effect of IRF-1 on mitochondrial depolarization and oxidative stress in A549 cells, we overexpressed IRF-1. Western blotting showed that IRF-1 was upregulated after transfection with an IRF-1 overexpression lentiviral vector (Fig. 4A). IRF-1 overexpression disrupted mitochondrial homeostasis and oxidative stress, as evidenced by increased ROS production and upregulated mitochondrial depolarization compared with the control (Fig. 4B, C). IRF-1 overexpression also promoted apoptosis (Fig. 4D) and led to ATP depletion, SOD consumption, and MDA accumulation in A549 cells (Fig. 4E–G).

### IRF-1 overexpression promotes apoptotic cell death and inhibits autophagy

**Fig. 5:**
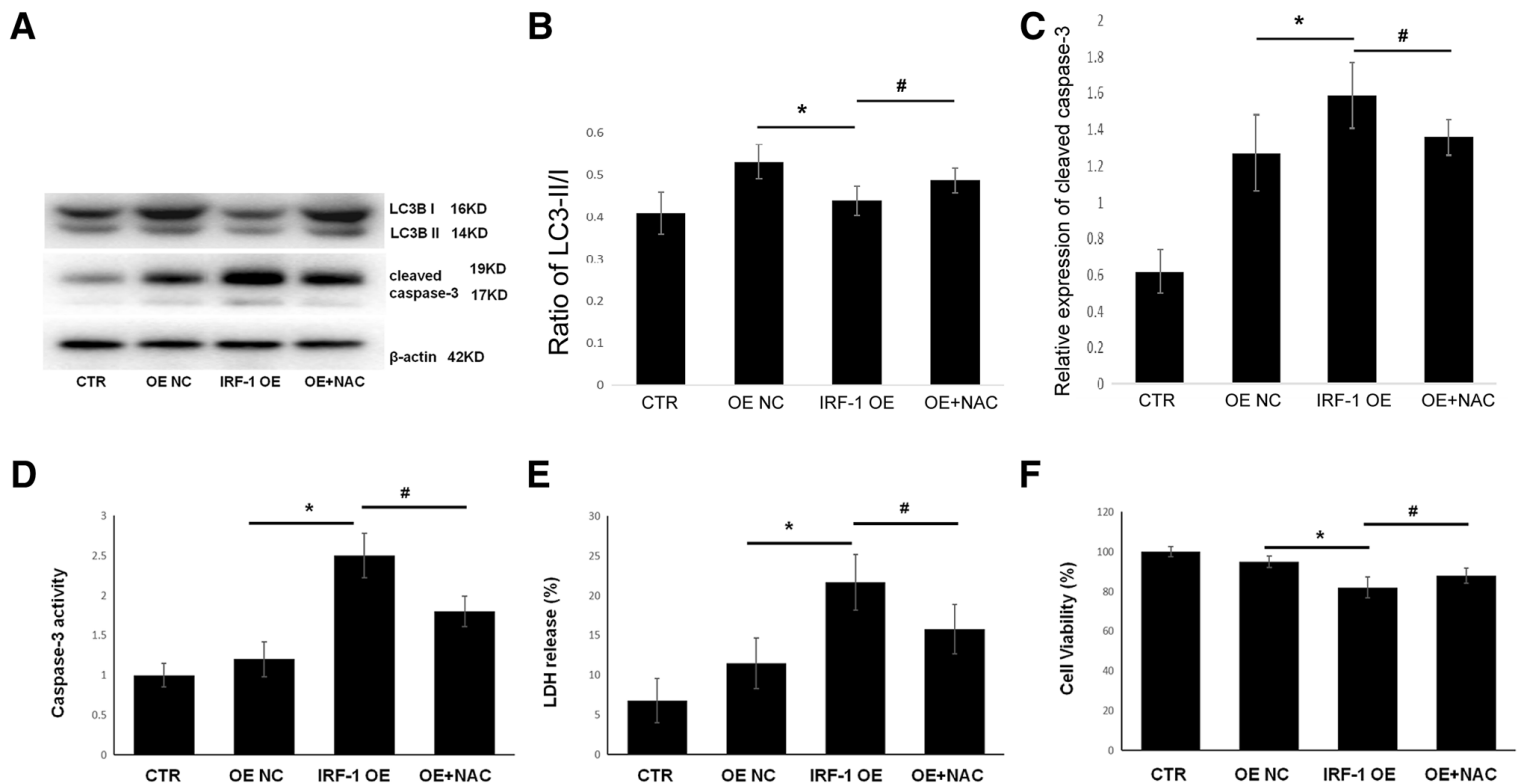
IRF-1 overexpression promotes apoptosis and inhibits autophagy. **A**–**C** Western blotting protein bands and corresponding quantitative analysis show the expression of cleaved caspase 3 and LC3-I/II; **D** Caspase 3 activity as determined by Caspase 3 Activity Assay Kit; **E** LDH detection using LDH release reagent; **F** Cell viability as determined by MTT assay. *P < 0.05 vs. OE NC, #*P* < 0.05 vs. NAC pretreatment. CTR, control; NC, negative control; OE, overexpression; NAC, *N*-acetylcysteine

We further investigated the effects of IRF-1 overexpression in A549 cells and found that it led to reduced conversion rate of LC3B-I/II in A549 cells (Fig. 5A, B), indicating an inhibitory effect of IRF-1 on autophagy. In addition, overexpression of IRF-1 increased cleaved-caspase 3 levels (Fig. 5A, C) and increased caspase 3 activity (Fig. 5D), indicating that IRF-1 overexpression promoted apoptosis. Overexpression of IRF-1 also increased LDH release (Fig. 5E) and decreased cell viability (Fig. 5F) in A549 cells. Interestingly, treatment with 5 mM *N*-acetylcysteine (NAC) alleviated IRF-1-mediated apoptosis and inhibition of autophagy (Fig. 5A–F). Taken together, these results suggest that IRF-1 might enhance the effect of cisplatin on A549 cells by inducing apoptosis, partially through an ROS-dependent pathway.

### Knockdown of IRF-1 protects against cisplatin-induced mitochondrial depolarization and oxidative stress

**Fig. 6:**
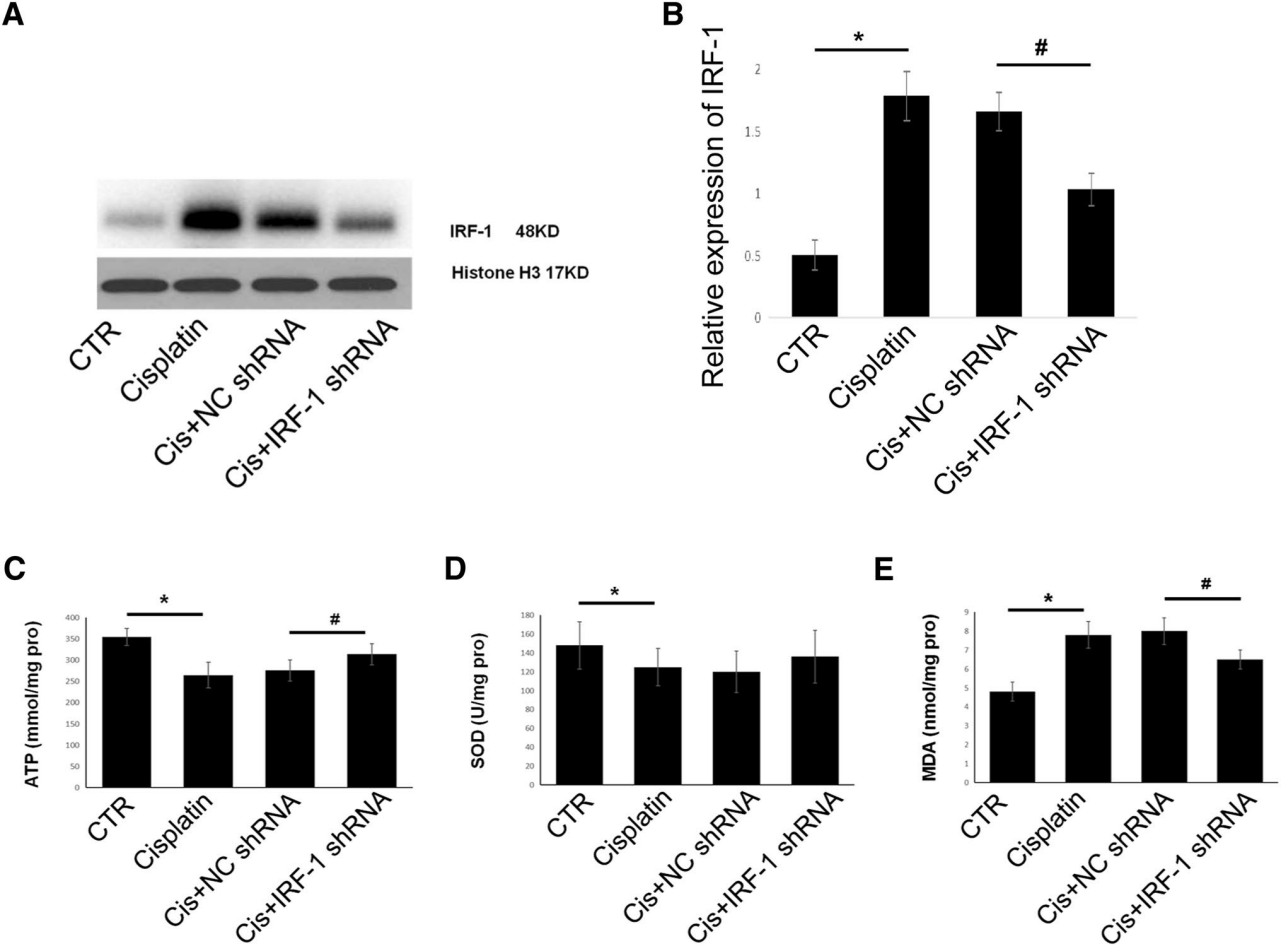
Knockdown of IRF-1 protects against cisplatin-induced mitochondrial depolarization and oxidative stress. **A**, **B** Western blotting protein bands and corresponding quantitative analysis show the expression of IRF-1 in each group; the levels of ATP (**C**), SOD (**D**), and MDA (**E**) as determined using respective test kits. *P < 0.05 vs. the control, #*P* < 0.05 vs. NC shRNA. CTR, control; NC, negative control

The expression of IRF-1 was significantly upregulated in cisplatin-treated A549 cells. Transfection of A549 cells with IRF-1 shRNA decreased the expression of IRF-1 (Fig. 6A, B). We then studied the effect of IRF-1 knockdown on cisplatin-induced mitochondrial depolarization and oxidative stress in A549 cells by measuring ATP, MDA, and SOD levels. The results showed that IRF-1 knockdown significantly reduced cisplatin-induced ATP consumption (Fig. 6C) and MDA accumulation (Fig. 6E), and although the difference was not statistically significant, SOD consumption showed a improvement trend (Fig. 6D) following IRF-1 knockdown.

### Knockdown of IRF-1 reduces cisplatin-induced apoptosis and promotes autophagy

**Fig. 7:**
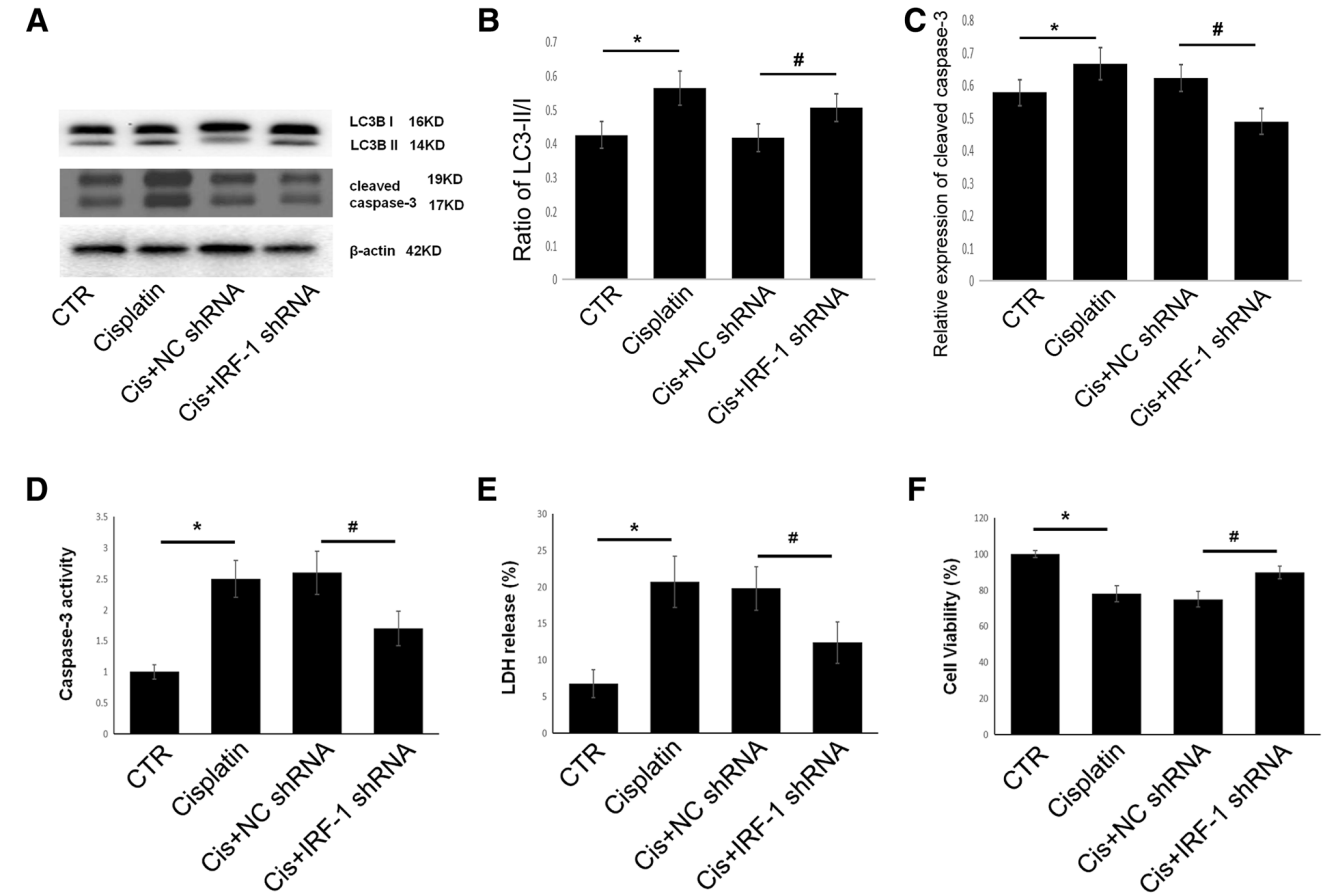
Knockdown of IRF-1 reduces cisplatin-induced apoptosis and promotes autophagy. **A**–**C** Western blotting proteins bands and corresponding quantitative analysis show the expression of cleaved caspase 3 and LC3-I/II; D, caspase 3 activity as determined by caspase 3 activity assay kit; **E** LDH as detected by LDH release reagent; **F** cell viability as determined by MTT assay. *P < 0.05 vs. the control group, #*P* < 0.05 vs. NC shRNA. CTR, control; NC, negative control

Cisplatin-induced apoptosis and autophagy in A549 cells, as demonstrated by increased caspase 3 cleavage and decreased LC3B-I/II conversion (Fig. 7A–C). Transfection with IRF-1 shRNA decreased cleaved caspase 3 levels and increased LC3B-I/II conversion compared with the levels in NC shRNA transfected cells (Fig. 7A–C), indicating that knockdown of IRF-1 inhibited cisplatin-induced apoptosis and promoted autophagy. Cisplatin treatment increased caspase 3 activity (Fig. 7D) and LDH release (Fig. 7E) and decreased cell viability (Fig. 7F). In IRF-1 knockdown A549 cells, cisplatin-induced caspase 3 activity (Fig. 7D) and LDH release were decreased (Fig. 7E) and cell viability was increased (Fig. 7F) compared with the control group. Therefore, knockdown of IRF-1 reduced cisplatin-induced apoptotic cell death and promoted autophagy.

## Discussion

Studies have indicated that deletion or dysfunction of IRF-1 can induce tumor occurrence or progression in various cancers, including gastric cancer [17], breast cancer [30] and hepatocellular carcinoma [31]. IRF-1 was significantly lower in hepatocellular carcinoma tumors than in noncancerous tissues, and activation of IRF-1 by IFN-γ-induced PD-L1 expression in vitro [31]. IRF-1 has been implicated in the response of cancer cells to cisplatin. Pavan et al. showed that IRF-1 expression in ovarian cancer cells was significantly increased by cisplatin treatment and IRF-1 overexpression inhibited the transformed phenotype of tumor cells [32]. Yuan et al. reported that IRF-1 was depleted in gastric cancer and that multiple drug resistance could be reversed by activating IRF-1 expression using doxycycline [23]. Consistently, we found that IRF-1 was depleted in NSCLC and cisplatin-induced IRF-1 activation in A549 cells.

Cisplatin-induced apoptosis is important for its anti-tumor activity. Mitochondria regulate multiple cellular functions, including cellular energy conversion, metabolism, ROS production and the intrinsic apoptotic pathway [33, 34]. Disturbances in mitochondrial homeostasis are important for triggering apoptosis [35]. Previous studies have shown that oxidative stress is a mediator of cisplatin-induced apoptotic cell death and is closely related to chemoresistance [36]. In this study, we found that overexpression of IRF-1 promoted apoptotic cell death and induced mitochondrial depolarization and oxidative stress in A549 lung cancer cells. Our previous study revealed that cisplatin-induced apoptotic cell death and the autophagic response in A549 cells, but the upstream regulator was unknown [29]. In this study, we demonstrated IRF-1 activation as a possible upstream regulator and showed that an imbalance in mitochondrial homeostasis is the central cisplatin resistance mechanism.

IRF-1 has been implicated in the upregulation of stress-induced apoptosis and downregulation of invasive activity in different cancer cells [37]. IRF-1 mediated apoptosis after treatment with ionizing radiation or chemotherapy drugs [38]. Overexpression of IRF-1 enhanced the chemosensitivity of gastric cancer cells to 5-fluorouracil [39], and high expression of IRF-1 also improved the efficiency of cisplatin-mediated killing of ovarian [40] and pancreatic cancer cells [41]. Therefore, we speculated that IRF-1 may disrupt mitochondrial homeostasis, causing mitochondrial depolarization and oxidative stress to promote apoptotic cell death in A549 cells.

Autophagy is a defense and adaptive response and cytoprotective mechanism that is important for the quality control process that maintains mitochondrial homeostasis [42]. Mitochondrial dysfunction or disruption of mitochondrial homeostasis, such as a respiratory injury or membrane depolarization, and excessive ROS accumulation have been implicated in tumorigenesis and drug responsiveness in multiple tumors [43]. Autophagy improves the tolerance of tumor cells to unfavorable environments as well as their survival under cisplatin treatment [44], which is the mechanism driving cisplatin resistance; thus inhibition of autophagy may reverse cisplatin resistance. In lung cancer and other malignant tumors, it has been suggested that enhanced autophagy may be related to drug resistance [45, 46]. In this study, we found that IRF-1 could inhibit autophagy, which might explain the reversal of cisplatin resistance in A549 cells.

This study preliminarily proposed that IRF-1 might be a possible target for increasing the sensitivity to cisplatin therapy by regulating apoptosis, autophagy, and mitochondrial homeostasis, and further studies were needed to elucidate the underlying exact mechanism. For example, cisplatin-induced apoptosis has been recognized as an important mechanism for its anti-tumor activity. The pathways of apoptosis can be divided into endogenous mitochondrial pathway, endogenous endoplasmic reticulum pathway and exogenous death receptor pathway [47]. In terms of endogenous mitochondrial apoptotic pathway, when the mitochondrial membrane potential decreases, the mitochondrial membrane permeability increases, and the pro-apoptotic factors in the mitochondria are released into the cytoplasm. After cytochrome C is released from mitochondrial into the cell, it interacts with apoptotic protease activating factor-1 (APAF-1) and forms apoptotic complex with the assistance of ATP and dATP, which forms caspase-9 holoenzyme by recruiting and activating pro-caspase-9. Caspase-9 holoenzyme further activates effector caspase 3 and caspase-7, initiating the Caspase cascade, which ultimately leads to apoptosis [48–50]. In this study, we found that IRF-1 disrupted mitochondrial homeostasis, causing mitochondrial depolarization to promote apoptotic cell death in A549 cells, and therefore we speculated that an endogenous mitochondrial apoptotic pathway was involved. However, only caspase 3 activity was detected, this speculation should be further confirmed by detecting other factors, such as caspase-9. Additionally, the regulatory mechanism of autophagy requires further study.

## Conclusions

**Fig. 8:**
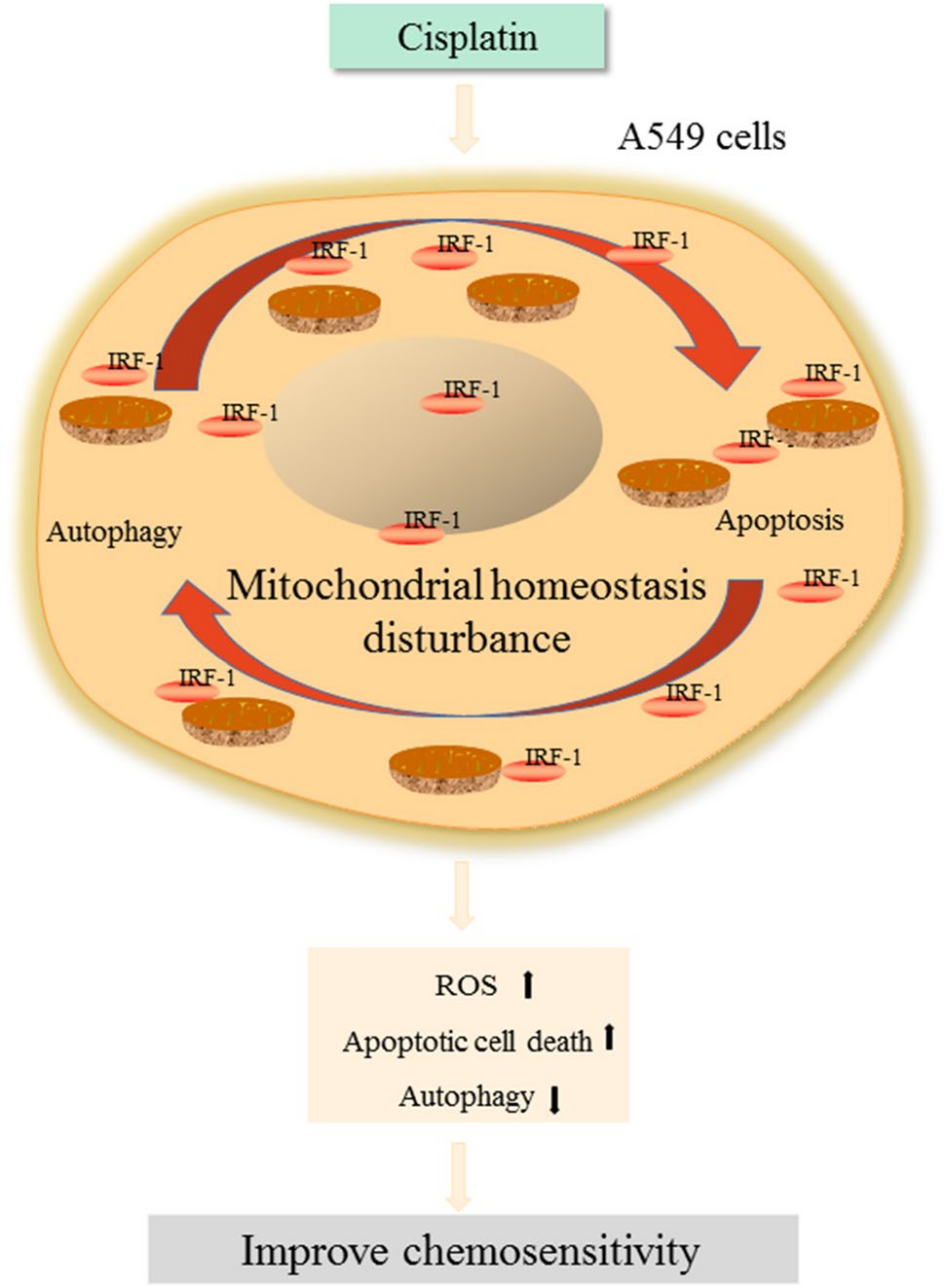
Schematic showing the biologic role of IRF-1 in lung cancer

In conclusion, we found that IRF-1 is depleted in NSCLC, and cisplatin induces IRF-1 activation. IRF-1 overexpression promotes apoptotic cell death, inhibits autophagy, and induces mitochondrial depolarization and oxidative stress. Our study is helpful in understanding the mechanisms by which cisplatin kills lung cancer cells and provides further compelling evidence that IRF-1 is involved in the anti-cancer effect of cisplatin by promoting apoptosis and inhibiting autophagy to disturb mitochondrial homeostasis (Fig. 8). These results suggest IRF-1 as a possible target for increasing the sensitivity to cisplatin therapy by regulating apoptosis, autophagy, and mitochondrial homeostasis. Our data also provide novel mechanistic insights into IRF-1 as an effector of cisplatin and the possibility of enhancing chemotherapy sensitivity by upregulating IRF-1 expression.

## Data Availability

The raw data, supporting the conclusions of this manuscript, will be made available by contacting the corresponding author.
